# Supplementary material for: Iterative improvement in the automatic modular design of robot swarms
Source: PeerJ Comput Sci. 2020 Dec 7;6:e322. doi: 10.7717/peerj-cs.322 (PMC7924708; doi:10.7717/peerj-cs.322)
Supplement: Supplemental Information 3 [file peerj-cs-06-322-s003.zip › argos3/doc/api/standalone/a00619_source.html]

ARGoS: plugins/simulator/physics\_engines/pointmass3d/pointmass3d\_box\_model.cpp Source File


- Main Page
- Related Pages
- Namespaces
- Classes
- Files

- File List
- File Members

# plugins/simulator/physics\_engines/pointmass3d/pointmass3d\_box\_model.cpp

Go to the documentation of this file.

```
00001 
00007 #include "pointmass3d_box_model.h"
00008 #include <argos3/core/utility/logging/argos_log.h>
00009 #include <argos3/core/utility/math/box.h>
00010 #include <argos3/core/simulator/simulator.h>
00011 #include <argos3/core/simulator/space/space.h>
00012 #include <argos3/plugins/simulator/physics_engines/pointmass3d/pointmass3d_engine.h>
00013 
00014 namespace argos {
00015 
00016    CPointMass3DBoxModel::CPointMass3DBoxModel(CPointMass3DEngine& c_engine,
00017                                               CBoxEntity& c_box) :
00018       CPointMass3DModel(c_engine, c_box.GetEmbodiedEntity()),
00019       m_cBoxEntity(c_box) {}
00020 
00021    /****************************************/
00022    /****************************************/
00023 
00024    void CPointMass3DBoxModel::CalculateBoundingBox() {
00025       CVector3 cHalfSize = 0.5f * m_cBoxEntity.GetSize();
00026       GetBoundingBox().MinCorner.Set(
00027          GetEmbodiedEntity().GetOriginAnchor().Position.GetX() - cHalfSize.GetX(),
00028          GetEmbodiedEntity().GetOriginAnchor().Position.GetY() - cHalfSize.GetY(),
00029          GetEmbodiedEntity().GetOriginAnchor().Position.GetZ());
00030       GetBoundingBox().MaxCorner.Set(
00031          GetEmbodiedEntity().GetOriginAnchor().Position.GetX() + cHalfSize.GetX(),
00032          GetEmbodiedEntity().GetOriginAnchor().Position.GetY() + cHalfSize.GetY(),
00033          GetEmbodiedEntity().GetOriginAnchor().Position.GetZ() + m_cBoxEntity.GetSize().GetZ());
00034    }
00035 
00036    /****************************************/
00037    /****************************************/
00038 
00039    bool CPointMass3DBoxModel::CheckIntersectionWithRay(Real& f_t_on_ray,
00040                                                        const CRay3& c_ray) const {
00041       CBox m_cShape(m_cBoxEntity.GetSize(),
00042                     GetEmbodiedEntity().GetOriginAnchor().Position,
00043                     GetEmbodiedEntity().GetOriginAnchor().Orientation);
00044       return m_cShape.Intersects(f_t_on_ray, c_ray);
00045    }
00046    
00047    /****************************************/
00048    /****************************************/
00049 
00050    REGISTER_STANDARD_POINTMASS3D_OPERATIONS_ON_ENTITY(CBoxEntity, CPointMass3DBoxModel);
00051 
00052    /****************************************/
00053    /****************************************/
00054 
00055 }
```

---

Generated on 10 Jul 2018 for ARGoS by 
 1.6.1
